# Accurate Classification of Minor Leaks in Primary Loop Systems Using a Multi-Scale Attention Temporal Convolutional Autoencoder

**DOI:** 10.3390/s26165289

**Published:** 2026-08-20

**Authors:** Jinghua Yang, Xiaohua Yang, Jie Liu, Zhuoran Xu, Guorui Huang

**Affiliations:** 1 School of Nuclear Science and Technology, University of South China, Hengyang 421001, China; 2 Hunan Engineering Research Center of Software Evaluation and Testing for Intellectual Equipment, Hengyang 421001, China; 3 Key Laboratory of Advanced Nuclear Energy Design and Safety, Ministry of Education, Hengyang 421001, China; 4 School of Computer Science, University of South China, Hengyang 421001, China

**Keywords:** fault category, reactor coolant system, autoencoder, zero-shot localization

## Abstract

Minor leaks in the reactor coolant system (RCS) can provide early warning of degradation that may progress towards safety-relevant failures in nuclear power plants. Their timely localization remains difficult because they generate weak thermal-hydraulic perturbations and labeled leak data are rarely available. Here, we present MATCA-TM, a physics-informed zero-shot framework that combines a Multi-scale Attention Temporal Convolutional Autoencoder (MATCA) with theoretical response-template matching for early leak localization. The framework learns multivariate temporal dependencies solely from normal operating data, enabling reconstruction residuals to highlight subtle anomalies caused by leaks. Physics-informed templates constructed from thermal-hydraulic response characteristics then provide a physically motivated basis for localizing leak sources. On the II + CPR1000 simulation platform, which represents a CPR1000 pressurized water reactor model, MATCA-TM achieved 65.3% localization accuracy for an equivalent leakage diameter of 6.7 × 10^−3^ cm and outperformed the evaluated machine learning and deep learning baselines under the same protocol. By combining data-driven representation learning with domain physics, MATCA-TM provides a physics-informed, simulation-validated approach to diagnosing incipient RCS leaks and a basis for further validation under plant-relevant operating conditions.

## 1. Introduction

The reactor coolant system (RCS) of a pressurized water reactor performs essential functions, including core heat removal, pressure-boundary maintenance, and energy transport [1,2]. Its operating condition is closely related to the safety and economic performance of nuclear power plants (NPPs). Minor leaks [3] typically occur during the early stages of coolant-system degradation and are characterized by low leak rates and slow fault evolution. The resulting changes in pressure, temperature, flow rate, and water level can be masked by measurement noise, control actions, and normal operating fluctuations [4,5]. If leakage is not identified early, it may develop during plant operation, increasing the risk of equipment failure and unplanned shutdowns. Early diagnosis therefore requires both anomaly detection and accurate localization of the leak to support maintenance planning and operational decisions.

Building on the success of deep learning in industrial anomaly detection and fault diagnosis, recent studies have extended these methods to leakage detection and localization in nuclear power plants (NPPs) [6]. Obra and Miwa [7] analyzed 428 labeled loss-of-coolant accident (LOCA) scenarios simulated using RELAP/SCDAPSIM and achieved 94–99% accuracy in classifying break locations for rupture extents ranging from 1% to 99%. Zhang et al. [8] reported an overall classification accuracy of 98.24% for hot leg LOCA scenarios in a CPR1000 plant simulated using the Personal Computer Transient Analyzer (PCTRAN), with equivalent break diameters of 7.98 and 10.70 cm. Qian and Liu [9] investigated leakage source localization using PCTRAN-PWR3LP Version 6.0.1, and achieved accuracies of 86.4% and 83.3% for equivalent break diameters of 3.57 cm and 5.05 cm, respectively, with labels from only 2% of the target-domain samples. These studies show that supervised models can distinguish leakage scenarios when representative labeled data is available. However, labeled minor leak samples are difficult to obtain because actual leakage events are rare and their thermal-hydraulic responses are weak [10,11]. Similar transient behaviors at different leakage locations further complicate fine-grained localization when only a limited number of samples are available.

Learning from normal operating data offers a potential solution for fault-scarce scenarios and has been explored in nuclear power applications. Zhou et al. [12] proposed DWCAN (Double Weighting of positive and negative samples for Clustering by Adopting Neighbors), a neighborhood-enhanced contrastive learning method that learns discriminative representations from unlabeled fault data to recognize unknown fault patterns. Song et al. [13] applied conventional machine learning (ML) methods, including K-means and Gaussian mixture models, to unsupervised clustering of multichannel monitoring signals. Wang et al. [14] developed STGCN (Spatiotemporal Graph Convolutional Networks), which models spatiotemporal relationships among monitoring variables to localize anomalies without requiring fault samples. Most existing unsupervised methods, however, classify samples primarily based on statistical characteristics or feature-space distances. They do not explicitly encode constraints imposed by the underlying fault mechanisms. When minor leak signals are weak, and responses from different locations substantially overlap, data-driven features alone may not provide stable localization or reveal the physical differences among leakage locations. A key challenge is therefore to incorporate thermal-hydraulic prior knowledge into leakage localization without requiring labeled leakage samples.

During normal reactor coolant system operation, reactor coolant pumps drive the primary coolant through the reactor core, hot legs, steam generators, and cold legs. Under mass, energy, and momentum conservation, along with neutronic feedback, monitored variables exhibit stable multivariate coupling. Although minor leaks at different locations may produce only small overall signal changes, the associated disturbance paths through the pressure boundary, flow field, heat-transfer process, and monitored variables are not identical. Location-dependent differences can therefore remain in the multivariate response patterns [15].

We therefore propose MATCA-TM (Multi-scale Attention Temporal Convolutional Autoencoder-based Template Matching), a physics-informed zero-shot method for minor-leak localization. The method combines a Multi-scale Attention Temporal Convolutional Autoencoder (MATCA) [16] with physics-informed template matching of theoretical responses. MATCA-TM learns multivariate temporal coupling characteristics from normal operating data and uses reconstruction residuals to highlight minor leak-induced changes. To distinguish highly similar responses, it constructs physics-informed theoretical response templates based on the thermal-hydraulic propagation mechanisms associated with different leakage locations, providing an effective solution for early, intelligent diagnosis of pressurized water reactor coolant systems.

The main contributions of this study are threefold:

(1) We formulate minor leak localization as a physics-informed zero-shot inference problem based on location-dependent thermal-hydraulic response mechanisms.
(2) We propose MATCA-TM, which integrates multiscale temporal anomaly representation with physics-informed theoretical response-template matching to localize minor leaks without using labeled leak samples during model training.
(3) We evaluate MATCA-TM under multiple leakage locations and severity levels, demonstrating its effectiveness in localizing minor leaks under weak-leak conditions.

## 2. Methods

The RCS removes heat from the reactor core and transfers it through the primary coolant. Under normal operating conditions, the coolant circulates through the reactor core, hot legs, steam generators, and cold legs, driven by the reactor coolant pumps. Accordingly, pressure, flow rate, temperature, reactor power, and water level are coupled rather than independent, with their relationships governed by mass, energy, and momentum conservation, as well as reactivity feedback [17,18,19,20]. A leakage event perturbs the local mass balance and pressure boundary conditions, resulting in changes in flow redistribution, momentum transfer, energy transport, and heat transfer. These perturbations propagate through the RCS along location-dependent paths, leading to abnormal conditions that manifest as changes in both individual variables and multivariate dynamic response patterns.

**Figure 1:**
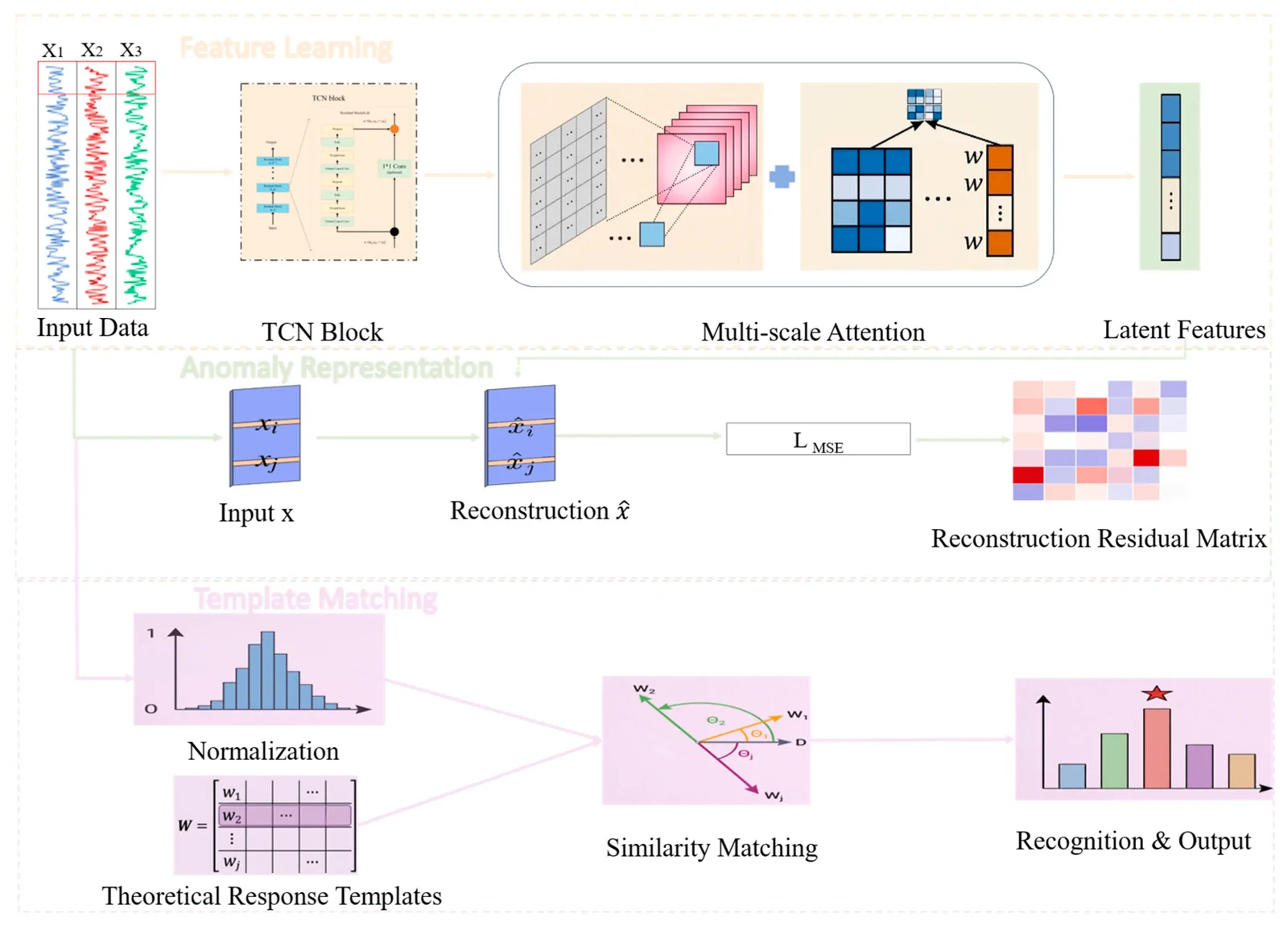
The flowchart of MATCA-TM.

A low leak rate, slow parameter evolution, and weak early-stage responses characterize minor leakage. During the initial stage, the reactor protection and safety injection systems are typically not activated, while the chemical and volume control system may compensate for coolant inventory loss by adjusting the charging flow rate. These control actions, together with measurement noise and normal operating fluctuations, can partially mask changes in primary-system pressure, pressurizer level, coolant flow rate, and coolant temperature. Therefore, the magnitude of an individual-variable deviation or reconstruction error alone is insufficient for reliable leak localization. Nevertheless, leakage at different locations enters the RCS through different physical positions and propagates along different thermal-hydraulic paths. Consequently, cold-leg, hot-leg, and steam-generator tube leaks produce different multivariate response patterns, even when their overall signal deviations are weak and highly similar. These location-dependent response patterns provide the physical basis for subsequent theoretical template matching. The overall workflow of the proposed MATCA-TM method is illustrated in Figure 1.

### 2.1. Normal Operating Condition Feature Learning 

MATCA-TM uses MATCA as its backbone network to learn multivariate temporal coupling relationships among monitored RCS parameters, establishing a reference model for subsequent anomaly identification.

First, multivariate monitoring data are transformed into temporal matrices $X_{i}\in R^{T\times M}$ using a sliding window, where i denotes the i-th time window, T is the window length, and M is the number of monitored variables. The encoder combines dilated causal convolutions in the Temporal Convolutional Network (TCN) with residual connections to capture long-term temporal dependencies. Multi-scale convolutional kernels and a channel attention mechanism are integrated into a multi-scale attention module. This module integrates information across temporal scales and emphasizes variables sensitive to abnormal operating conditions. The encoder therefore produces an attention-enhanced latent representation $Z_{att}$, which captures the dynamic coupling characteristics of the RCS. The decoder uses a symmetric architecture to reconstruct the input sequence. To reduce overfitting to abnormal variations, attention is applied only in the encoder. The reconstruction loss $L_{MSE}$ is defined as the mean squared error (MSE) for normal operating conditions:

(1) $$L_{MSE}=\frac{1}{N}\sum_{i=1}^{N}\frac{1}{TM}\sum_{t=1}^{T}\sum_{m=1}^{M}{\left(x_{i,t,m}-{\hat{x}}_{i,t,m}\right)}^{2}$$

where $x_{i,t}^{m}$ and ${\hat{x}}_{i,t}^{m}$ are the true and reconstructed values of the m-th monitored variable at time step t in the i-th time window, respectively, and N is the number of training samples.

After training, the model establishes a multivariate coupling baseline for normal operating conditions. For a given testing sample, the reconstruction residual matrix is defined as:

(2) $$E_{i}=X_{i}-{\hat{X}}_{i}$$

where $X_{i}$ and ${\hat{X}}_{i}$ denote the input and reconstructed sequences of the i-th test sample, respectively. The reconstruction error is then compared with a control threshold $\alpha_{\mathrm{KDE}}$, which is estimated from the reconstruction errors of normal validation samples. Samples with reconstruction errors exceeding $\alpha_{\mathrm{KDE}}$ are identified as anomalous and subsequently passed to the physics-informed template matching module for leakage-type identification, whereas samples below the threshold are classified as normal and excluded from further classification. 

### 2.2. Construction of Physics-Informed Theoretical Response Templates

MATCA-TM constructs physics-informed theoretical response templates for different leakage locations based on the conservation laws and coupled transport processes governing thermal-hydraulic behavior in the RCS. A leakage perturbs the local mass balance and pressure boundary conditions, thereby affecting flow redistribution, momentum transfer, energy transport, heat transfer, and associated control-system and neutronic feedback. Based on these location-dependent physical response characteristics, theoretical templates are constructed to characterize the expected response pattern associated with each leakage location. For an anomalous sample identified by the reconstruction-error threshold, its observed response pattern is then compared with these physics-informed templates to determine the most consistent leakage location.

The template weights were not calculated analytically from the governing equations. Instead, domain experts assigned them based on the physical relevance of each monitored variable to the corresponding leakage location. These weights represent the relative importance of the monitored variables in characterizing location-specific leakage responses.

To match these templates with the anomaly responses extracted from test samples, the reconstruction residuals must first be converted into a comparable representation. Direct comparisons of reconstruction residuals are unreliable because the monitored variables have different physical units and different normal reconstruction-error levels. The reconstruction-residual matrix for each test sample is therefore standardized using residual statistics estimated from normal operating data. Let the mean and standard deviation for the m-th monitored variable under normal operation be denoted by $\mu_{m}^{0}$ and $\sigma_{m}^{0}$, respectively:

(3) $$D_{i,t,m}=\frac{E_{i,t,m}-\mu_{m}^{0}}{\sigma_{m}^{0}+\varepsilon }$$

where ε is a small positive constant used to avoid division by zero. The standardized residuals are then aggregated over time to form a residual-response vector $r_{i}$ that characterizes the spatial pattern of multivariate abnormalities:

(4) $$r_{i,m}=\frac{1}{T}\sum_{t=1}^{T}\left|D_{i,t,m}\right|\;\;\;m=1,2,\ldots ,M$$

For the k-th representative leakage type, a physics-informed theoretical response template $W_{k}$ is constructed from expert-assessed relative response weights for the corresponding leakage location:

(5) $$W_{k}=\left(w_{k,1},w_{k,2},\ldots ,w_{k,M}\right)$$

where $w_{k,m}$ represents the relative response weight of the m-th monitored variable under the k-th leakage type. 

To reduce the influence of leakage magnitude and fault-evolution stage on anomaly amplitudes, the residual response vector and the physics-informed theoretical response template are further normalized using the $L_{2}$ norm:

(6) $${\tilde{r}}_{i}=\frac{r_{i}}{{\left\|r_{i}\right\|}_{2}}$$

(7) $${\tilde{W}}_{k}=\frac{W_{k}}{{\left\|W_{k}\right\|}_{2}}$$

This normalization preserves relative response patterns among key monitored variables while removing differences in absolute magnitude. The leakage types are represented by a set of prior physics-informed theoretical response templates, denoted by $\left\{{\tilde{W}}_{1},{\tilde{W}}_{2},\ldots ,{\tilde{W}}_{K}\right\}$, where K is the number of predefined leakage categories. These templates provide reference patterns for subsequent leakage-type identification.

### 2.3. Leakage Type Identification Based on Similarity Matching

For each anomalous test sample, MATCA-TM compares its normalized residual-response vector ${\tilde{r}}_{i}$ with all physics-informed theoretical response templates using cosine similarity, which quantifies the agreement between their multivariate residual patterns. For the k-th leakage type, the matching score is defined as:

(8) $$S_{i,k}={\tilde{r}}_{i}^{T}{\tilde{W}}_{k}\;\;\;\;\;\;k=1,2,\ldots ,K$$

The maximum similarity score is then defined as:

(9) $$S_{i}^{max}=\underset{k\in \left\{1,2,\ldots ,K\right\}}{\max}S_{i,k}$$

The similarity threshold $\tau_{sim}$ is determined from normal validation data without using labeled leakage samples. Specifically, the maximum similarity score $S_{i}^{max}$ is calculated for each normal validation sample against all predefined templates, and $\tau_{sim}$ is set to the 99th percentile of the resulting distribution. The instantaneous identification result for an individual time window is then determined as:

(10) $${\hat{y}}_{i}=\left\{\begin{array}{cc} \arg\underset{k\in \left\{1,2,\ldots ,K\right\}}{\max}S_{i,k}, & S_{i}^{max}\geq \tau_{sim},\\ Unknown, & S_{i}^{max}<\tau_{sim}. \end{array}\right.$$

An anomalous sample is assigned to a predefined leakage category only when its maximum similarity exceeds $\tau_{sim}$. Otherwise, it is rejected as an unknown anomalous condition. This rejection criterion prevents forced assignment and is not intended as a separate supervised unknown-fault classification task.

The instantaneous labels obtained from consecutive time windows are further aggregated using ${\hat{y}}_{i}$, a temporal voting mechanism to reduce the influence of measurement noise and local fluctuations. Because minor leakage events are temporally continuous, this mechanism promotes consistent decisions across adjacent windows. Let the predicted category sequence within a decision window of length L be $\left\{{\hat{y}}_{i-L+1},\ldots ,{\hat{y}}_{i}\right\}$. The final decision is determined as:

(11) $${\hat{Y}}_{i}=\mathrm{mode}\left({\hat{y}}_{i-L+1},\ldots ,{\hat{y}}_{i}\right)$$

where $\mathrm{mode}\left(\cdot \right)$ denotes the majority-voting operator, and Unknown is treated as a valid decision label.

Thus, MATCA-TM enables zero-shot leakage localization by matching reconstruction residuals to be predefined, physically motivated response templates, without using labeled leakage samples during model training. For reproducibility, the MATCA-TM workflow is summarized as pseudocode in Algorithm A1 in Appendix A.

## 3. Experiments

### 3.1. Experimental Design

To evaluate MATCA-TM for minor leak localization, we conducted simulation experiments using the II + CPR1000 pressurized-water-reactor simulator [21,22,23]. The platform provides 108 monitoring parameters intended to represent a range of reactor operating conditions. We selected three representative leakage scenarios for validation: cold-leg leakage (cold leg), hot-leg leakage (hot leg), and steam-generator tube leakage (steam). To examine performance across leakage severities, we designed three leakage conditions with equivalent diameters of $6.7\times 10^{-1}$ cm, $6.7\times 10^{-2}$ cm, and $6.7\times 10^{-3}$ cm. The experiments were evaluated based on leakage severity and leakage location.

The training set contained 24 h of continuous normal-operation data, including routine load regulation and natural process fluctuations. The test set consisted of a continuous sequence: 2 h of normal operation followed by 6 days of minor-leak data. At 1 Hz, the normal and leakage stages contained 7200 and 518,400 samples, respectively, for a total of 525,600 samples per complete sequence. The initial 2-h period was used to assess false alarms without leakage. The 6-day observation window was selected to provide a sufficiently long period for evaluating the temporal evolution and localization performance of minor leaks. 

We selected three representative baselines from different technical paradigms: DWCAN, STGCN, and ML. Each method was implemented as described in its original study and, when necessary, adapted to the three-class minor-leak localization task. For a fair comparison, all methods used the same data preprocessing, training and test partition, input variables, sampling frequency, normalization procedure, and temporal-window strategy. To accommodate different input/output structures, we modified only the interfaces, while keeping the core architectures and principal loss functions unchanged. The hyperparameters for each baseline were primarily set according to the original study. Missing or incompatible parameters were adjusted using the normal-operation validation set and a consistent model-selection criterion.

All models were evaluated on the same hardware platform, comprising an Intel Core i9 CPU (Intel Corporation, Santa Clara, CA, USA), an NVIDIA GeForce RTX 4090 GPU (NVIDIA Corporation, Santa Clara, CA, USA), and 32 GB of RAM (Kingston, Fountain Valley, CA, USA). The same data partition, preprocessing procedure, and evaluation protocol were used for all methods. Localization performance was assessed using accuracy (ACC), precision, recall, F1 score, and false-positive rate (FPR). Davies–Bouldin Index (DBI) and Silhouette Coefficient (SC) were used to assess feature-space compactness and separability. Each experiment was repeated 10 times to reduce the influence of random initialization, and results are reported as mean ± standard deviation.

### 3.2. Data 

The experimental data were generated with a nuclear steam supply system (NSSS) simulator configured to represent the II + CPR1000 nuclear power plant. The simulator supports leakage cases with equivalent break diameters ranging from $6.7\times 10^{-3}$ cm to $1.386\times 10^{1}$ cm. This study focuses on minor leaks in the RCS, including the hot leg, cold leg, and steam-generator heat-transfer tubes. All data were sampled at 1 Hz.

Following the partitioning strategy in Section 3.1, the training set contained 86,400 samples, corresponding to 24 h of normal operation. The complete test sequence contained 525,600 samples, including 7200 normal-operation samples from the first 2 h and 518,400 minor leak samples from the subsequent 6 days. The sequence covered multiple leakage types and severities. To approximate noise, Gaussian white noise with an intensity of 0.5% was added to all time-series data. Leakage samples were not used for feature selection; instead, only the multivariate statistics and temporal coupling relationships of normal operating data were used. analysis of variance (ANOVA) and Pearson correlation analysis were applied to assess variable relevance and identify redundant variables, thereby reducing the input dimensionality while retaining variables that better characterize the system response. Twenty sensitive variables were selected from the initial 108 monitored variables as model input. Response templates were then constructed from expert-assessed relative weights based on the thermal-hydraulic relevance and disturbance-propagation characteristics of these variables for different leakage locations (Table 1).

### 3.3. Evaluation Metric

We evaluated localization performance and feature separability using classification and clustering validity metrics. The classification metrics included accuracy (ACC), precision, recall, F1 score, and false-positive rate (FPR), while the clustering validity metrics included the Davies–Bouldin Index (DBI) and Silhouette Coefficient (SC). The metric definitions are given below:

(12) $$ACC=\frac{TP+TN}{TP+TN+FP+FN}$$

(13) $$Precision=\frac{TP}{TP+FP}$$

(14) $$Recall=\frac{TP}{TP+FN}$$

(15) $$F1\;score=\frac{2\times TP}{2\times TP+FP+FN}$$

(16) $$FPR=\frac{FP}{FP+TN}$$

(17) $$DBI=\frac{1}{K}\sum_{k=1}^{K}\underset{j\neq k}{max}\left(\frac{\sigma_{k}+\sigma_{j}}{d(c_{k},c_{j})}\right)$$

(18) $$SC=\frac{1}{N}\sum_{i=1}^{N}\frac{b(i)-a(i)}{max\{a(i),b(i)\}}$$

For each leakage class, the class under evaluation was treated as the positive class, while the remaining classes were treated as the negative class. Accordingly, TP, TN, FP, and FN denote the numbers of true-positive, true-negative, false-positive, and false-negative samples, respectively. K denotes the total number of leakage classes. $\sigma_{k}$ is the average intra-cluster dispersion of the k-th cluster, and $d(c_{k},c_{j})$ is the Euclidean distance between the centroids of the k-th and j-th clusters. For the Silhouette Coefficient, a(i) denotes the average distance between sample i and all other samples in the same cluster, whereas b(i) denotes the average distance between sample i and all samples in its nearest neighboring cluster.

### 3.4. Parameter Settings 

The MATCA-TM hyperparameters are summarized in Table 2. Because MATCA-TM uses the MATCA backbone, the temporal-convolution and optimization parameters were kept consistent with those used for MATCA. The final reconstruction-error and similarity thresholds were determined based on the sensitivity analysis presented below. All experiments were conducted on the same workstation equipped with an Intel Core i9 CPU (Intel Corporation, Santa Clara, CA, USA), NVIDIA RTX 4090 GPU (NVIDIA Corporation, Santa Clara, CA, USA), and 32 GB RAM (Kingston, Fountain Valley, CA, USA) for consistency.

The threshold analysis shows that the combination of $\alpha_{\mathrm{KDE}}$ = 95.0% and $\gamma_{\mathrm{sim}}$ = 99.0% provides the selected trade-off, with an accuracy of 0.682 and an FPR of 0.355, as shown in Table 3 Compared with stricter reconstruction-error thresholds, the lower $\alpha_{\mathrm{KDE}}$ increases sensitivity to weak leakage signals. The high similarity threshold ($\gamma_{\mathrm{sim}}$) helps prevent low-confidence samples from being assigned to known leakage categories. Conversely, overly strict reconstruction-error thresholds reduce false alarms but may suppress weak leakage responses. These results support the selected setting as a trade-off between false-alarm suppression and leakage-type classification accuracy under this evaluation protocol.

## 4. Results

**Figure 2:**
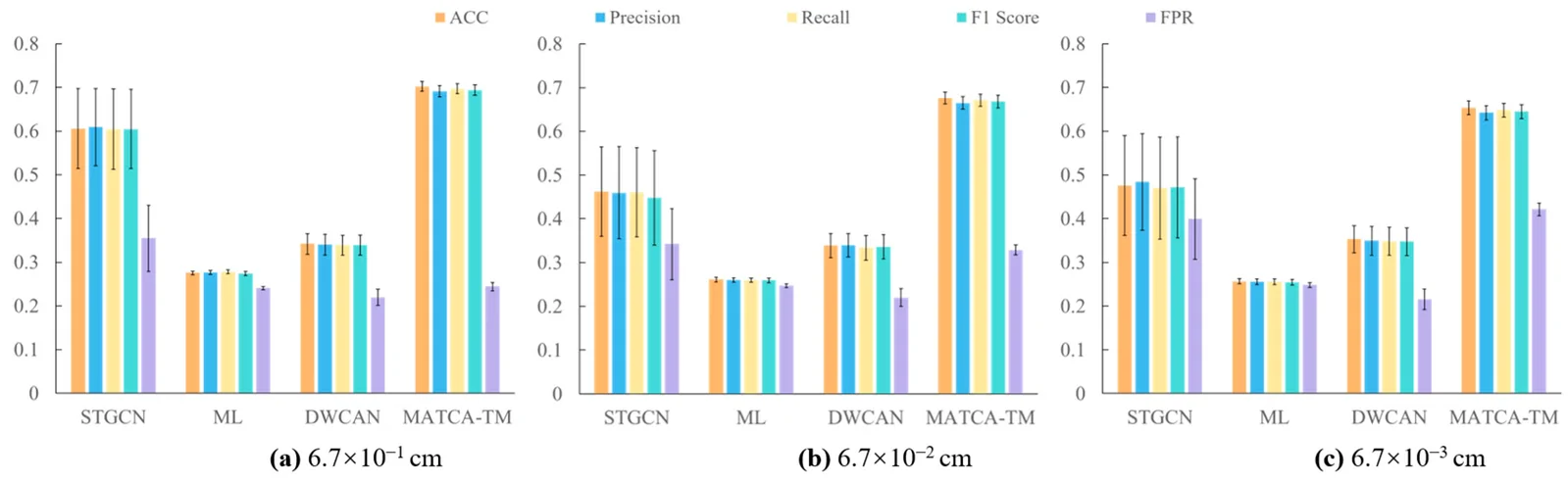
Detection results of STGCN, ML, DWCAN and MATCA-TM.

**Figure 3:**
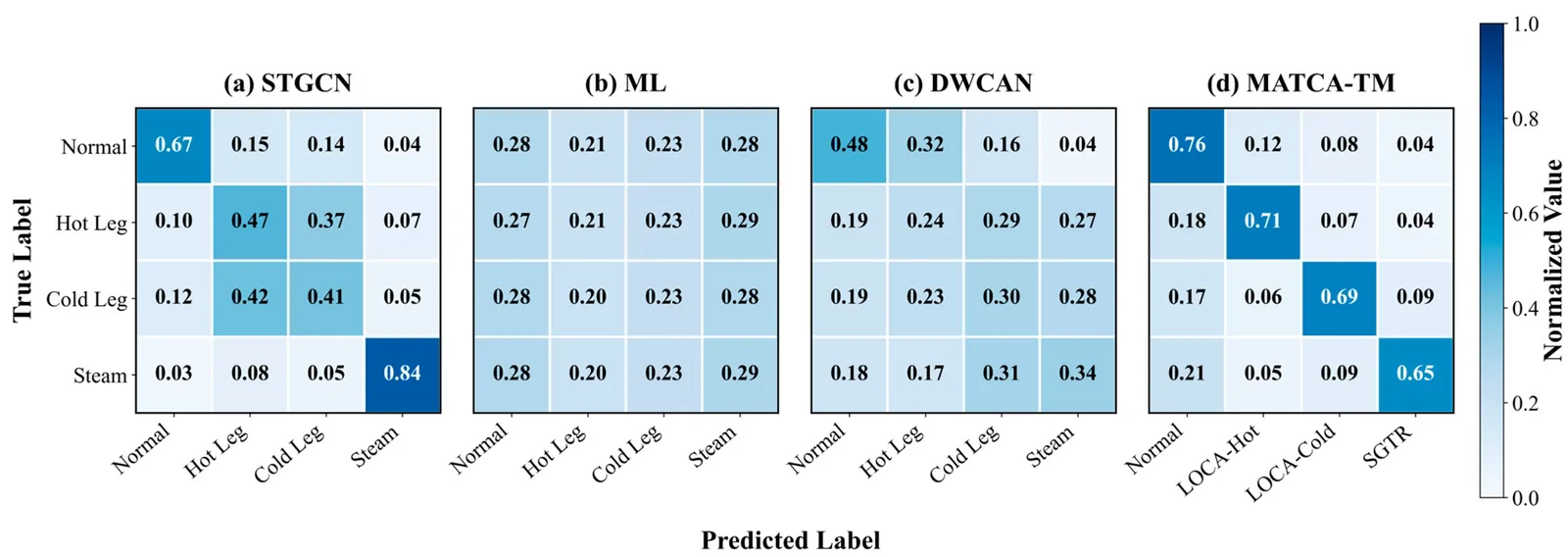
Confusion matrices for $6.7\times 10^{-1}\;cm$ under four experimental cases.

**Figure 4:**
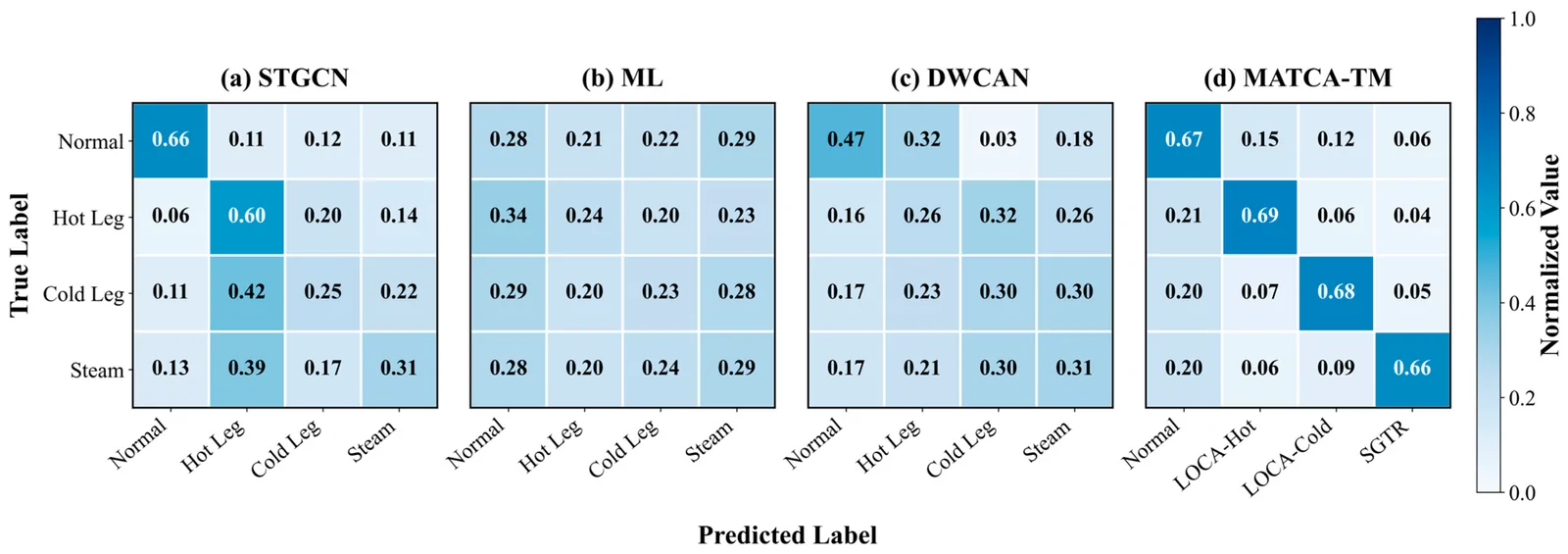
Confusion matrices for $6.7\times 10^{-2}\;cm$ under four experimental cases.

**Figure 5:**
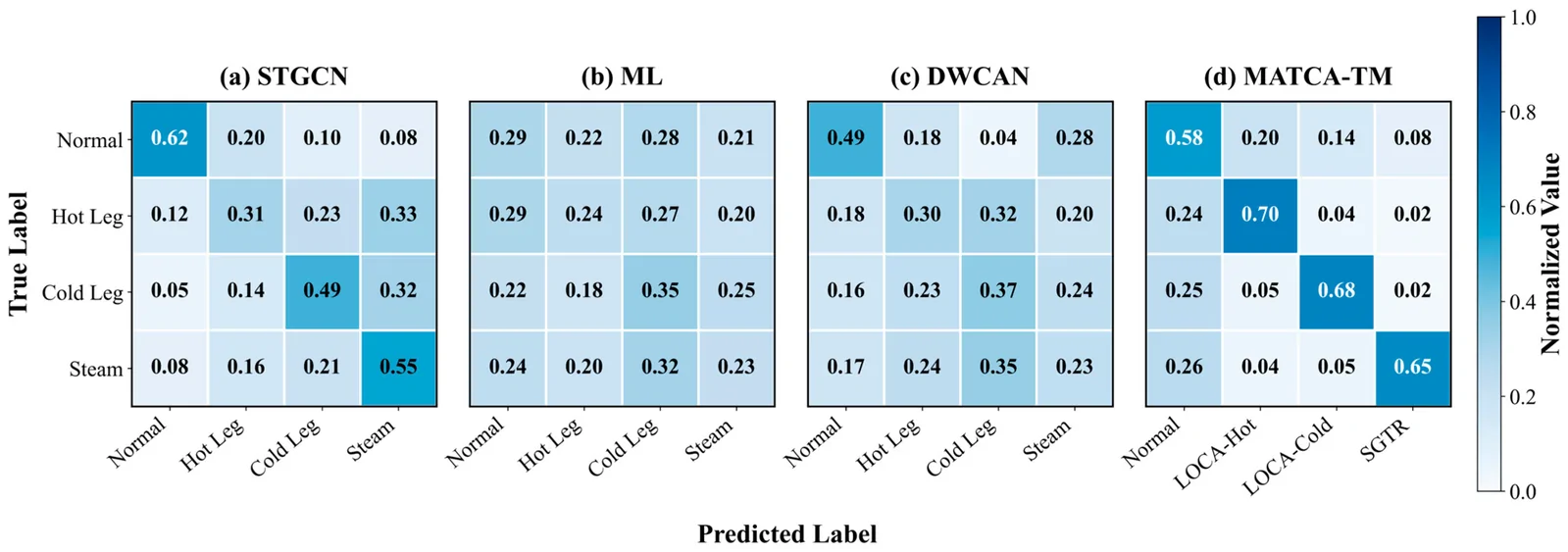
Confusion matrices for $6.7\times 10^{-3}\;cm$ under four experimental cases.

Figure 2, Figure 3, Figure 4 and Figure 5 compare MATCA-TM with ML, DWCAN, and STGCN under different leakage conditions. As the equivalent leakage diameter decreases from $6.7\times 10^{-1}$ cm to $6.7\times 10^{-3}$ cm, the differences between leakage-type responses become less pronounced, leading to performance degradation across all evaluated methods.

ML relies mainly on statistical features and clustering structure and does not explicitly model long-term temporal dependencies. It therefore has difficulty distinguishing leakage types when leak-induced signals are weak. DWCAN improves latent-feature separation via contrastive learning and outperforms ML in the evaluated setting. Its discrimination, nevertheless, remains primarily distance-based, and different leakage categories still overlap in feature space when leak-induced signals are weak. STGCN uses structural relationships among variables to learn spatiotemporal features and performs well for relatively large leaks. As leak-induced responses weaken, the learned feature distributions overlap more strongly, reducing separability.

In contrast, MATCA-TM localizes by matching spatial patterns in multivariate residual responses to physics-informed theoretical templates. It showed more consistent performance across the evaluated leakage intensities. The relatively small standard deviations across 10 independent runs indicate limited run-to-run variability, though the variability increased slightly as leakage severity decreased. At an equivalent leakage diameter of $6.7\times 10^{-3}$ cm, MATCA-TM achieved 65.3% localization accuracy, outperforming ML, DWCAN, and STGCN in this simulation study. Misclassifications mainly occurred between leakage conditions with similar response patterns. Under weak leakage conditions, the reduced differences in local responses among leakage locations weakened the discriminative power of template matching.

**Figure 6:**
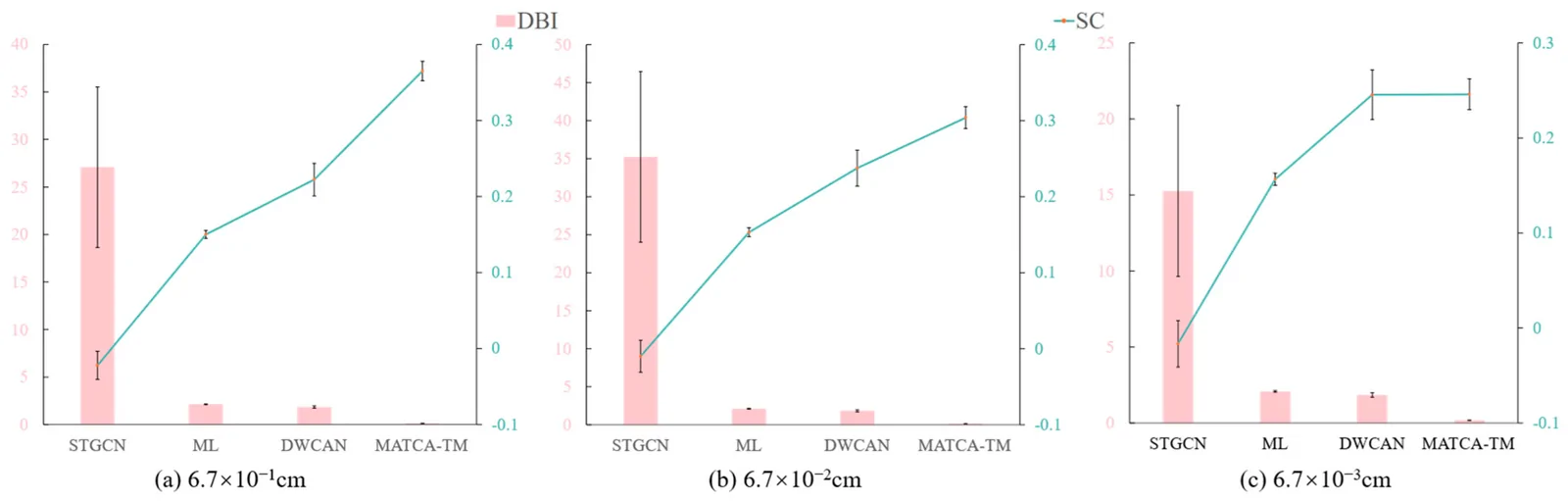
Clustering performance of different methods.

**Figure 7:**
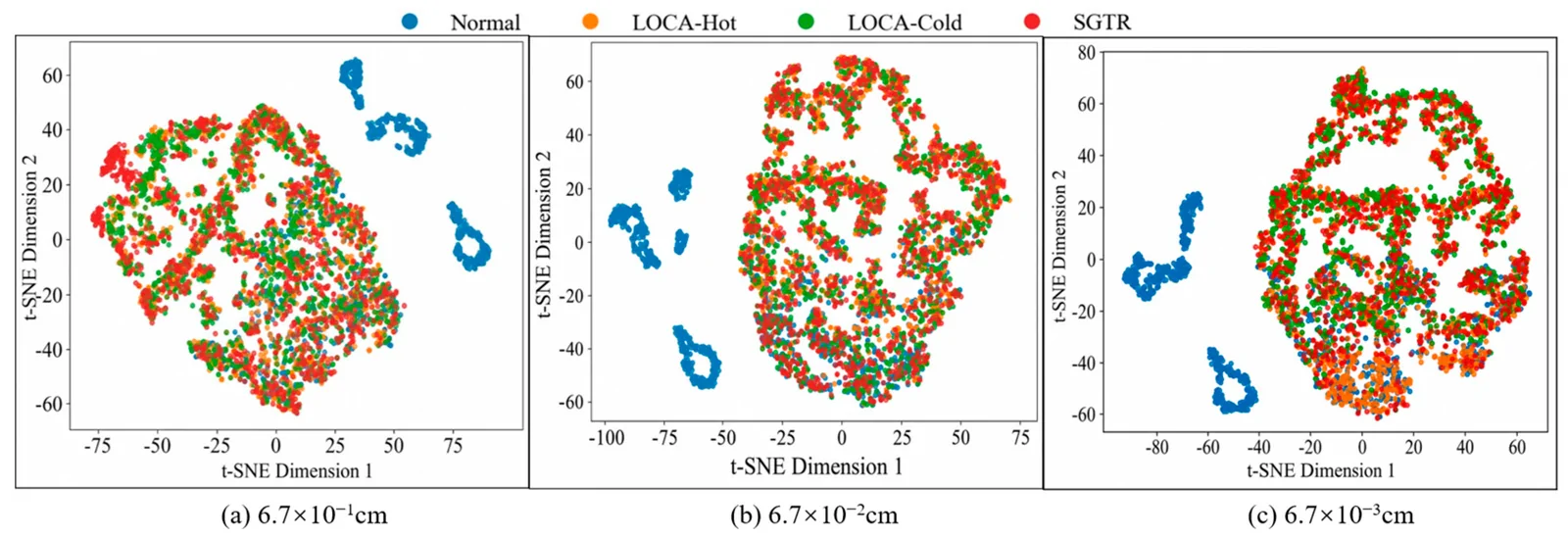
T-SNE visualization of features extracted by STGCN method under various fault severity levels.

**Figure 8:**
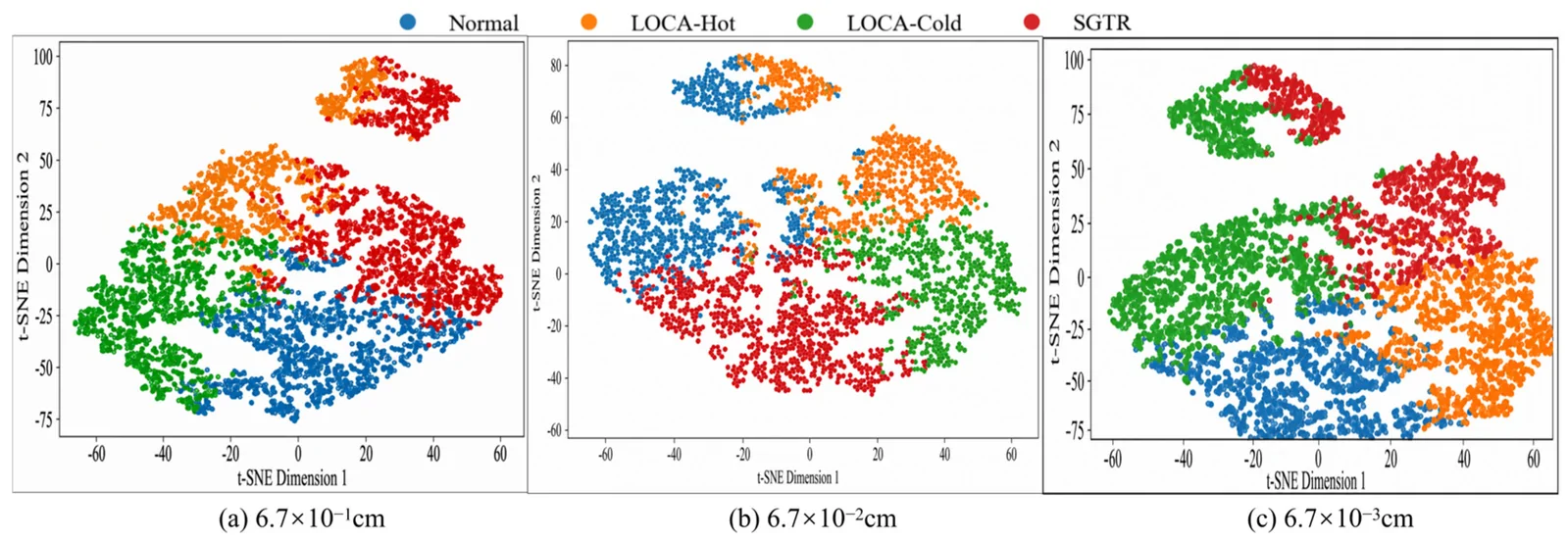
T-SNE visualization of features extracted by ML method under various fault severity levels.

**Figure 9:**
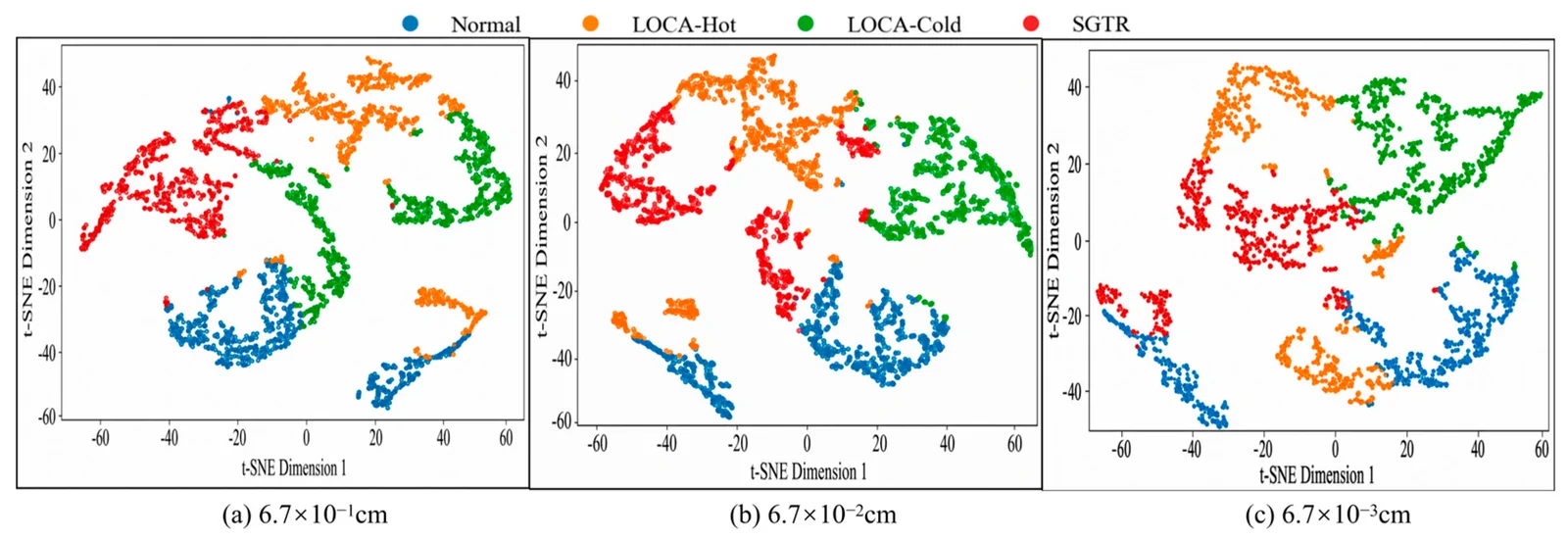
T-SNE visualization of features extracted by DWCAN method under various fault severity levels.

**Figure 10:**
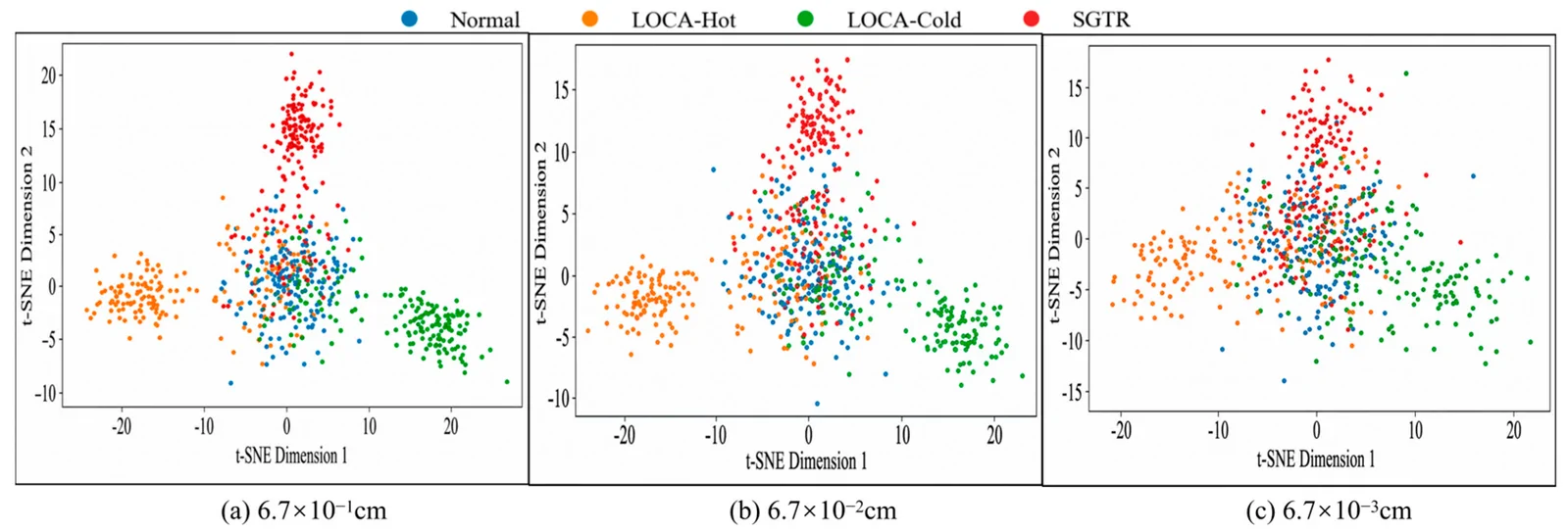
T-SNE visualization of features extracted by MATCA-TM method under various fault severity levels.

Figure 6, Figure 7, Figure 8, Figure 9 and Figure 10 present clustering performance and t-distributed Stochastic Neighbor Embedding (t-SNE) feature distributions of the evaluated methods. In Figure 4a,b, the features learned by STGCN and ML are highly intertwined, leading to blurred class boundaries under weak leakage conditions. DWCAN achieves better clustering, but some cluster overlaps remain. MATCA-TM produces more compact within-class clusters and better between-class separation than the evaluated baselines. The relatively small standard deviations of DBI and SC across repeated runs indicate that these clustering patterns were consistent across different random initializations. Overall, these results suggest that combining reconstruction residuals with physics-informed response templates can improve the separation of leakage types under the tested conditions.

## 5. Discussion

Minor leak signals in the RCS are often masked by measurement noise and operational fluctuations, making early leak localization particularly challenging when labeled leakage samples are unavailable and the response patterns of different leakage locations overlap. MATCA-TM addresses this challenge by combining temporal feature learning from normal-operation data with physics-informed response templates. The model is trained exclusively on normal-operation data, and anomalous samples are subsequently localized by matching their response patterns with location-specific templates derived from the thermal-hydraulic mechanisms of the RCS.

To clarify the relative advantages and limitations of MATCA-TM, Table 4 compares it with ML, DWCAN, and STGCN across detection performance, computational complexity, minimum detectable leakage diameter, diagnostic mechanism, label dependence, and interpretability. Under the evaluated conditions, MATCA-TM achieved a favorable balance between detection performance, computational requirements, and interpretability. ML and DWCAN achieved F1 scores below 35%, while STGCN achieved F1 scores below 60% under weaker leakage conditions. In contrast, MATCA-TM maintained an F1 score above 60% for a leakage diameter as small as $6.7\times 10^{-3}$ cm under the criterion adopted in Table 4.

In terms of computational complexity, STGCN constructs its graph through pairwise ARIMA fitting among n monitored variables, resulting in an O(n^2^) preprocessing step. MATCA-TM does not require this pairwise ARIMA-based graph construction. During online monitoring, its additional template-matching operation depends primarily on the number of predefined leakage-location templates, while the main computational cost remains associated with the MATCA feature extraction and reconstruction network. This design may therefore facilitate continuous monitoring.

Several limitations should be acknowledged. Under extremely weak leakage conditions, the leak-induced response may approach the noise floor, making it more difficult to distinguish response patterns across different leakage locations. In addition, although the II + CPR1000 simulator captures the major thermal-hydraulic characteristics relevant to the investigated leakage scenarios, practical factors such as sensor drift and long-term operational variability are beyond the scope of the present study. Future work will focus on validating MATCA-TM under broader operating conditions and more realistic data while improving its localization capability under extremely weak leakage conditions.

## 6. Conclusions

We propose MATCA-TM, a physics-informed zero-shot method for minor leak localization. The normalized residual-response patterns are then matched with predefined physics-informed response templates to localize leakage. MATCA learns temporal coupling patterns from normal operating data and uses reconstruction residuals to highlight subtle leak-induced changes. The method then matches spatial residual patterns to predefined response templates to localize leaks without using labeled leakage samples during model training.

On the II + CPR1000 simulation platform, MATCA-TM achieved a localization accuracy of 65.3% for weak leakage with an equivalent diameter as small as $6.7\times 10^{-3}$ cm, and outperformed ML, DWCAN, and STGCN under the evaluated protocol. These results support the method’s potential for simulation-based studies of minor-leak localization. Future work will focus on validating the framework under broader operating conditions and more realistic data, particularly under extremely weak leakage conditions.

## Figures and Tables

**Table 1:** Main monitored variables and expert-assessed relative response weights for different leakage locations.

Number	Parameters	Expert-Assessed Relative Weight		
		Hot Leg	Cold Leg	SGTR
1	Nuclear Power	0.3	0.3	0.2
2	Electric Power	0.3	0.3	0.4
3	Primary Coolant Circuit Water Level	1	1	0.9
4	Opening Degree of #1 Spray Valve	0	0	0
5	Opening Degree of #2 Spray Valve	0.1	0.1	0.1
6	Charging Flowrate	0.8	0.8	0.9
7	Surge Line Flowrate	0.9	0.8	0.7
8	Main Feedwater Temperature of Steam Generator A	0.2	0.2	0.4
9	Main Feedwater Temperature of Steam Generator B	0.2	0.2	0.4
10	Main Feedwater Temperature of Steam Generator C	0.2	0.2	0.4
11	Reactivity	0.6	0.8	0.5
12	Normalized Rotational Frequency of #3 Feed Pump	0.3	0.3	0.9
13	Outlet Temperature of #3 Feed Pump	0.1	0.1	0.2
14	Deaerator Pressure	0.3	0.3	0.5
15	Main Steam Header Pressure	0.6	0.6	0.8
16	Cavitation Head	0.6	0.8	0.4
17	Deaeration Extraction Steam Flowrate	0.2	0.2	0.3
18	Liquid Level of #1 Condenser	0.1	0.1	0.7
19	Pressure of #2 Condenser	0.2	0.2	0.5
20	Containment Pressure	0.9	0.8	0

**Table 2:** Parameters of the MATCA-TM model.

Hyperparameter	Value
Dilation rates	(1, 2, 4)
Number of TCN stacks	4
Number of filters	64
Kernel size (TCN)	3
Dropout rate	0.05
Conv1D filter size	32
Multi-scale kernel sizes	(3, 6, 9)
Conv activation	ReLU
Optimizer	Adam
Learning rate	1 × 10^−3^
Batch size	16
Epochs	200
Early stopping patience	5
Control limit method	KDE
$\alpha_{\mathrm{KDE}}$	0.95
$\gamma_{\mathrm{sim}}$	0.99
Noise level	0.005
Time steps	5

**Table 3:** Sensitivity analysis of the reconstruction-error threshold and similarity threshold.

	$\alpha_{\mathrm{KDE}}$	$\gamma_{\mathrm{sim}}$	Accuracy	FPR
1	95.00%	99.00%	68.20%	35.50%
2	95.00%	97.50%	67.50%	38.00%
3	97.50%	99.00%	66.80%	36.50%
4	97.50%	97.50%	66.00%	39.50%
5	99.00%	99.00%	65.30%	42.10%

**Table 4:** Comparative analysis of MATCA-TM and representative methods.

	ML	DWCAN	STGCN	MATCA-TM
Detection efficiency	F1: 25.49–27.39% FPR: 24.09–24.81%	F1: 33.59–34.73% FPR: 21.51–21.98%	F1: 44.83–60.49% FPR: 34.21–39.94%	F1: 64.5–69.4% FPR: 24.4–42.1%
Computational complexity	O(n^2^)	O(n)	O(n^2^)	O(n)
Smallest detectable leakage diameter (F1 > 60%)	Not achieved	Not achieved	6.7 × 10^−1^ cm	6.7 × 10^−3^ cm
Diagnostic mechanism	K-means-GMM-SVM	Contrastive clustering	Reconstruction-graph modeling	Physics-informed response template matching
Labeled fault samples	No	No	No	No
Interpretability	Medium	Low	High	High

## Data Availability

The data presented in this study are available upon request from the corresponding authors.

## Appendix A

**Algorithm A1:** MATCA-TM Diagnostic Algorithm
**Input:** Normal training data *X*_train_^0^. Normal validation data *X*_val_^0^. Test monitoring sequence *X*_test_. Physics-informed theoretical response templates {*W*_1_, *W*_2_, …, *W_K_*}. Window length *T*; voting window length *L*. Confidence level *α*. **Output:** Final diagnostic result *Y_i_*. 1: Train the MATCA reconstruction model using only normal training samples. 2: Reconstruct the normal validation samples and calculate reconstruction errors. 3: Determine the reconstruction-error control limit *τ*_rec_ using KDE at confidence level *α*. 4: Calculate the normal residual statistics *μ_m_*^0^ and *σ_m_*^0^ for each monitored variable. 5: Normalize all physics-informed theoretical response templates *W_k_* using the L2 norm. 6: Calculate the maximum template similarity scores of normal validation samples. 7: Set the similarity threshold *τ*_sim_ as the *α*-percentile of these scores. 8: for each test window *X_i_* do 9: Reconstruct *X_i_* using the trained MATCA model. 10: Calculate the reconstruction error *q_i_*. 11: if *q_i_* ≤ *τ*_rec_ then 12: *y_i_* = Normal 13: else 14: Calculate the reconstruction residual *E_i_* = *X_i_* − ${\hat{X}}_{i}$. 15: Standardize *E_i_* using *μ_m_*^0^ and *σ_m_*^0^. 16: Aggregate the standardized residuals into a residual response vector *r_i_*. 17: Normalize *r_i_* using the L2 norm. 18: Calculate cosine similarities *S_i_*_,*k*_ between *r_i_* and each template *W_k_*. 19: Find *S_i_^max^* and the corresponding leakage type *k*^*^. 20: if *S_i_^max^* ≥ *τ*_sim_ then 21: *y_i_* = *k*^*^ 22: else 23: *y_i_* = Unknown 24: end if 25: end if 26: end for 27: Apply majority voting to {*y_i_*_−*L*+1_, …, *y_i_*}. 28: Return the final diagnostic result *Y_i_*.
